# In Vitro Studies to Evaluate the Intestinal Permeation of an Ursodeoxycholic Acid-Conjugated Oligonucleotide for Duchenne Muscular Dystrophy Treatment

**DOI:** 10.3390/pharmaceutics16081023

**Published:** 2024-08-01

**Authors:** Marika Faiella, Giada Botti, Alessandro Dalpiaz, Lorenzo Gnudi, Aurélie Goyenvalle, Barbara Pavan, Daniela Perrone, Matteo Bovolenta, Elena Marchesi

**Affiliations:** 1 Department of Translational Medicine, University of Ferrara, 44121 Ferrara, Italy; fllmrk@unife.it (M.F.); bvlmtt@unife.it (M.B.); 2 Department of Chemical, Pharmaceutical and Agricultural Sciences, University of Ferrara, 44121 Ferrara, Italy; bttgdi@unife.it (G.B.); dla@unife.it (A.D.); mrclne@unife.it (E.M.); 3 Center for Translational Neurophysiology of Speech and Communication (CTNSC@UniFe), Italian Institute of Technology (IIT), 44121 Ferrara, Italy; 4 Department of Environmental and Prevention Sciences, University of Ferrara, 44121 Ferrara, Italy; lorenzo.gnudi@unife.it; 5 University Paris-Saclay, UVSQ, Inserm, END-ICAP, 78000 Versailles, France; aurelie.goyenvalle@uvsq.fr; 6 Department of Neuroscience and Rehabilitation—Section of Physiology, University of Ferrara, 44121 Ferrara, Italy

**Keywords:** Duchenne muscular dystrophy, antisense oligonucleotides, exon skipping, conjugated oligonucleotides, ursodeoxycholic acid, exosomes, co-culture, IEC-6 cells, DMD myotubes, intestinal permeation

## Abstract

Delivery represents a major hurdle to the clinical advancement of oligonucleotide therapeutics for the treatment of disorders such as Duchenne muscular dystrophy (DMD). In this preliminary study, we explored the ability of 2′-*O*-methyl-phosphorothioate antisense oligonucleotides (ASOs) conjugated with lipophilic ursodeoxycholic acid (UDCA) to permeate across intestinal barriers in vitro by a co-culture system of non-contacting IEC-6 cells and DMD myotubes, either alone or encapsulated in exosomes. UDCA was used to enhance the lipophilicity and membrane permeability of ASOs, potentially improving oral bioavailability. Exosomes were employed due to their biocompatibility and ability to deliver therapeutic cargo across biological barriers. Exon skipping was evaluated in the DMD myotubes to reveal the targeting efficiency. Exosomes extracted from milk and wild-type myotubes loaded with 5′-UDC-3′Cy3-ASO and seeded directly on DMD myotubes appear able to fuse to myotubes and induce exon skipping, up to ~20%. Permeation studies using the co-culture system were performed with 5′-UDC-3′Cy3-ASO 51 alone or loaded in milk-derived exosomes. In this setting, only gymnotic delivery induced significant levels of exon skipping (almost 30%) implying a possible role of the intestinal cells in enhancing delivery of ASOs. These results warrant further investigations to elucidate the delivery of ASOs by gymnosis or exosomes.

## 1. Introduction

Duchenne muscular dystrophy (DMD; OMIM #310200) is an X-linked recessive neuromuscular disorder characterized by the progressive loss of skeletal, respiratory, and cardiac muscles, followed by their replacement with fibrotic tissue after inflammation and necrosis [1]. DMD results from mutations in the dystrophin gene that induce frameshift and/or premature termination codons in the protein’s coding sequence, resulting in the loss of the dystrophin protein [2].

Despite the long-standing knowledge of the disease’s primary cause and the FDA approval of several oligonucleotide drugs [3], such as Exondys 51 (Eteplirsen 2016), Vyondys 53 (Golodirsen 2019), Viltepso (Viltolarsen 2020) and Amondys 45 (Casimersen 2021), and of the glucocorticoid Emflaza (Deflazacort 2017) [4] and nonsteroidal drug Duvyzat (Givinostat 2024) [5], there is no current cure for DMD [6,7,8].

Exon skipping, based on the use of antisense oligonucleotides (ASOs) able to modulate the splicing process and restore the dystrophin reading frame, has long been considered the most promising therapeutic approach to treat DMD [9,10,11]. Several chemically modified ASOs have been developed to improve their nuclease resistance, binding affinity to RNA, and ability to cross cell membranes and reduce their toxicity. These include 2′-*O*-methyl- and 2′-*O*-methoxyethyl-phosphorothioate (2′OMe PS and 2′-MOE PS), phosphorodiamidate morpholino oligomers (PMO), tricyclo-DNA (tc-DNA), stereopure and mixmer oligonucleotides [12,13,14]. Although encouraging improvements have been obtained in most cases, several limitations, including poor cellular uptake, endosomal entrapment and toxic dose constraints, remain to be solved [15,16,17,18,19].

**Figure 1:**
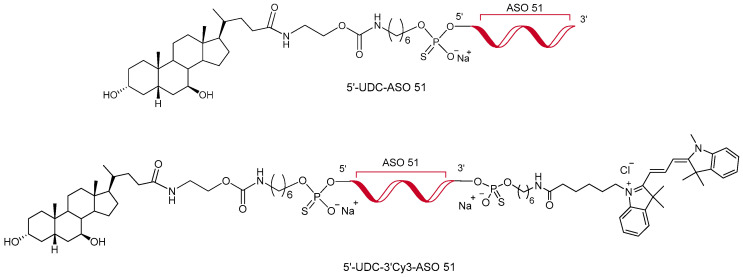
Chemical structure of 5′-UDC-ASO 51 [25] and 5′-UDC-3′Cy3-ASO 51.

The bioconjugation of appropriate functional molecules has recently appeared as a promising strategy to address several issues [20]. Among these, neutral lipophilic molecules, such as fatty acids, cholesterol, or squalene, have attracted considerable interest, since the ability of liposoluble moieties to facilitate cell membrane interactions enhanced the uptake and effectiveness of ASOs [20,21,22,23]. In this context, we recently reported the synthesis and the in vitro exon skipping properties of an ASO 51 conjugated at 3′ and/or 5′-end with a selection of lipophilic compounds [24,25]. Notably, the 2′OMe PS ASO, containing the sequence of the clinically evaluated Drisapersen [26,27,28], conjugated at 5′-end to ursodeoxycholic acid (UDCA) (named 5′-UDC-ASO 51, Figure 1), was found to be highly efficient in skipping the targeted human exon 51, when compared to the unconjugated control [25].

**Figure 2:**
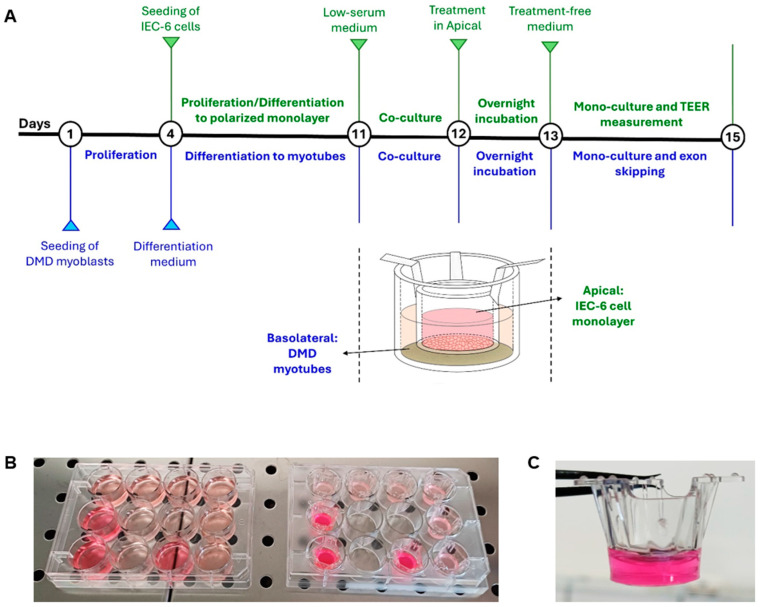
Experimental design. (**A**) Schematic illustration of the step-by-step experimental design utilized in the non-contact co-culture experiment. Treatment = incubation with 100 µM (about 400 µg) 5′-UDC-3′Cy3-ASO 51 as free compound (gymnosis) or loaded in MilkEXOs. (**B**,**C**) Non-contact co-culture methodology used with cell culture inserts, highlighting the technical setup and arrangement of the cell culture system; the photos were acquired after the overnight incubation of 5′-UDC-ASO 51 as free compound or loaded in MilkEXO.

In the present study, we aimed at evaluating the ability of ASO-51 and its conjugate 5′-UDC-ASO 51 to cross the intestinal barrier, which would open promising perspectives for oral administration of these compounds. In particular, the intestinal barrier was simulated in vitro by monolayers of normal epithelial small intestine IEC-6 cells of rat [29]. The polarized cell monolayers separate an apical from a basolateral compartment corresponding to the lumen facing domain and the blood-facing side of the monolayer, respectively (Figure 2B,C) [30].

This investigation was completed by evaluating the ability of 5′-UDC-ASO 51 to induce exon skipping in muscle cells after the intestinal IEC-6 monolayer crossing. To perform this analysis, a co-culture system of non-contacting IEC-6 cells and myotubes was set up. This system allows analysis of whether soluble or specific products secreted from IEC-6 cells, grown on the permeable membrane of commercial inserts, may affect the transport mechanisms and the internalization of ASOs in DMD myotubes, which were grown on the bottom of the well. To validate our system, the 5′-UDC-3′Cy3-ASO 51 has also been synthesized and exploited (Figure 1).

This preliminary study of the potential oral administration of ASOs was, moreover, accompanied by the investigation of an innovative formulation involving the emerging technology of exosomes (EXOs), which are membrane-bound extracellular vesicle particles produced by eukaryotic cells, measuring 30–150 nm [31,32]. Exosomes are identified as optimal natural carriers for the transport of macromolecules, such as lipids, proteins, nucleic acids, and microRNAs [18,33,34], and are emerging as promising tools in the treatment of neuromuscular disorders [35]. Furthermore, exosomes offer unique advantages, including high tissue specificity, customizable cargo, and reduced immunogenicity [36]. Exosomes offer several benefits over other delivery systems as viral vectors, and nanoparticles. They are naturally occurring, biocompatible, and less immunogenic, reducing the risk of adverse immune responses. Exosomes have an inherent ability to facilitate cellular uptake and deliver therapeutic cargo across biological barriers, enhancing the delivery efficiency of drugs and genetic material. However, their limitations include the complexity of the isolation and purification processes, potential for unintended cargo delivery, and scalability challenges for large-scale production. Despite these challenges, exosomes hold significant promise for targeted and efficient drug delivery. In the DMD context, EXOs have been implicated in both the exacerbation and mitigation of disease progression [37,38,39].

Our study aims to evaluate the possibility of using exosomes as natural vehicles for the delivery of 2′OMe PS antisense oligonucleotides ASO 51 and 5′-UDC-ASO 51 by testing the delivery and activity of the EXO–ASO complexes on muscle cells. In particular, a comparison between the free ASOs and EXO–ASO complexes is performed by evaluating their properties on muscle cells upon direct incubation or after crossing the intestinal IEC monolayer. The results of these studies reveal that our in vitro arrangement constitutes a valuable tool to evaluate the potential contribution of IEC-6 cells in the regulation of the exon skipping in DMD myotubes, highlighting the biological relevance of cell co-culture, as already reported [40].

## 2. Materials and Methods

### 2.1. Materials

High-performance liquid chromatography (HPLC)-grade acetonitrile (CH_3_CN) and triethylamine (TEA) were acquired from Carlo Erba Reagents S.A.S. (Milan, Italy). Merck Life Sciences Srl. (Milan, Italy) supplied the acetic acid glacial and dimethyl sulfoxide (DMSO). The Sartorius Arium^®^ Advance EDI (Sartorius Lab Instruments GmbH and Co., KG, Göttingen, Germany) system was used to obtain water (H_2_O) employed in the HPLC analysis. ThermoFisher Scientific (Milan, Italy) and Microtech (Naples, Italy) furnished Dulbecco’s modified Eagle’s medium (DMEM) with Glutamax, fetal bovine serum (FBS), penicillin/streptomycin, trypsin–EDTA, and Dulbecco’s phosphate buffered saline (DPBS). The sterile plastic material used for the cell culture was obtained from Thermo-Fisher Scientific (Milan, Italy) and Biosigma (Venice, Italy). Sabeu GmbH (Northeim, Germany) furnished the 12-well cellQART inserts. Merck Life Sciences Srl (Milan, Italy) supplied the rat small intestine IEC-6 cell line (RRID:CVCL_0343) in accordance with the terms and conditions of the supply of products from the Culture Collections of Public Health England (Culture Collections), including the European Collection of Authenticated Cell Cultures (ECACC). Reagents and solvents not mentioned were of analytical grade and obtained from Merck Life Sciences Srl. (Milan, Italy).

Human-derived skeletal muscle cell lines KM1328 and 8220 [24] were kindly provided by Professor Vincent Mouly (Institute of Myology, Paris, France) prepared by the Myoline platform. KM1328 were derived from a DMD patient with deletion of exon 52 (Δ52), whereas 8220 were derived from a healthy donor. The two cell lines were grown in Skeletal Muscle Growth Medium (Promocell, Heidelberg, Germany) and then differentiated into myotubes over a period of at least 7 days using Skeletal Muscle Differentiation Medium (Promocell, Heidelberg, Germany) with 2% horse serum, under incubation conditions of 37 °C and 5% CO_2_. For exosome purification purposes, 8220 cells were cultured with exosome-depleted serum and differentiated without serum.

### 2.2. Synthesis of Antisense Oligonucleotides

The synthesis of oligonucleotides was accomplished at 7 μmol scale employing ÄKTA oligopilot 10 PLUS (GE Healthcare, Milan, Italy) equipped with UNICORN software (version 5.10), following our previously reported protocols [24,25]. Briefly, 5′-UDC-ASO 51 was synthesized as previously reported [25], whereas 5′-UDC-3′Cy3-ASO 51 with 3′ modification was synthesized on Primer Support^TM^ C6 Amino Linker 200 (GE Healthcare, Milan, Italy) and Cy3-NHS (Lumiprobe, Hannover, Germany) conjugation was performed in solution following our post-synthetic approach [24]. Oligonucleotides were purified by reverse-phase High Performance Liquid Chromatography and the counterion exchange was performed by ion exchange chromatography.

### 2.3. HPLC Analysis

The High Performance Liquid Chromatography (HPLC) method was used to quantify both ASO 51 and 5′-UDC-ASO 51 using a modular system made of a LC-40D pump and SPD-M40 DAD detector from Shimadzu (Kyoto, Japan), completed with an injection valve consisting of a 20 µL sample loop (model 7725; Rheodyne, IDEX, Torrance, CA, USA). All analyses were conducted at room temperature using a Hypersil BDS C-18 column (5 µm, 150 mm × 4.6 mm i.d.) supplied by ThermoFisher Scientific SpA Italia Srl. (Milan, Italy) accessorized with a guard column made of the same material. The elution was performed using a mobile phase made of a mixture of 100 mM triethylammonium acetate (TEAA) and CH_3_CN in a ratio defined by a gradient profile with a flow rate of 1 mL/min; in particular, an isocratic elution using 10% (*v*/*v*) of CH_3_CN in the aqueous solvent was maintained for 3 min, followed by a linear gradient set to 60% (*v*/*v*) of CH_3_CN in the aqueous solvent within 10 min. Finally, a linear gradient allowed restoration of the initial mobile phase made of 10% (*v*/*v*) CH_3_CN in the aqueous solvent within 15 min, maintained for 5 min as isocratic elution to guarantee the re-equilibration of the system to the initial conditions. The chromatograms resulting from the analysis were displayed at 260 nm for both ASO 51 and 5′-UDC-ASO 51, whose retention times, in the described conditions, resulted as 7.7 min and 9.0 min, respectively. LabSolutions Software (version 5.110 in Windows 10, Shimadzu, Kyoto, Japan) was used to acquire and process data. Figures S1 and S2 reports representative chromatograms obtained by HPLC analysis of ASO 51 and 5′-UDC-ASO 51 at the described conditions. Repeated analysis of the same sample solution (10 µL, *n* = 6), which contained every single compound dissolved in DPBS at 5 µM, allowed evaluation of the chromatographic precision, represented by relative standard deviation (RSD) values of 0.89 and 0.86 for ASO 51 and 5′-UDC-ASO 51, respectively. Considering that solutions of each compound dissolved in DPBS in a range from 0.3 to 20 µM, calibration curves of peak areas versus concentration were generated and resulted as linear (*n* = 7, *r* ≥ 0.995, *p* < 0.001).

### 2.4. IEC-6 Cells’ Culture and Differentiation to Polarized Monolayers

Rat normal small intestine IEC-6 cells were seeded on T75 flasks and cultured in Dulbecco’s Modified Eagle’s Medium (DMEM) containing Glutamax, 10% Fetal Bovine Serum (FBS), 100 µg/mL penicillin and 100 U/mL streptomycin at 37 °C in a humidified atmosphere of 95%, with 5% of CO_2_. After two passages with trypsinization, confluent cells were seeded in 12-well cellQART cell culture inserts (Sabeu GmbH and Co. KG, Northeim, Germany) consisting of 1.0 μm pore size polyethylene terephthalate (PET) filter membranes, whose surface was 1.12 cm^2^. In particular, filters were pre-soaked for 24 h with 2 mL of fresh culture medium in the lower compartment (basolateral, B); then, the upper compartment (apical, A) received 400 μL of the diluted cells (8 × 10^4^ cells/insert). Cells were fed in apical and basolateral compartments every second day for about 7 days until the cell monolayer was fully confluent and, one day before starting the experiment, the medium was replaced on both sides of the monolayer by low-serum DMEM medium (1% FBS). After 24 h, the cell monolayers’ integrity was evaluated by measuring the transepithelial electrical resistance (TEER, Ω·cm^2^) using a voltmeter (Millicell–ERS; Millipore, Milan, Italy). The TEER values of cell monolayers, obtained by subtracting the background resistance of cell-free inserts from the resistance read across the insert with cells, reached at confluence a stable value of about 50 Ω·cm^2^ within eight days. In addition, phase contrast microscopy was used to monitor the homogeneity and integrity of the IEC-6 cell monolayer before the permeation studies.

### 2.5. MTT Assay for Evaluation of ASO 51, 5′-UDC-ASO 51 and 5′-UDC-3′Cy3-ASO 51 Toxicity on IEC-6 Cells

The potential toxicity of ASO 51, 5′-UDC-ASO 51 or 5′-UDC-3′Cy3-ASO 51 in IEC-6 cells, seeded at a density of 5 × 10^3^ cells/well in 96-well plate, was assayed with the 3-(4,5-dimethylthiazol-2-yl)-2,5-diphenyltetrazolium (MTT) test. Specifically, cells were incubated overnight at 37 °C in a humidified 5% of CO_2_ atmosphere at 37 °C with increasing concentrations of (i) ASO 51 or 5′-UDC-ASO 51 (10 20, 30, 50, 100 µM) or (ii) 5′-UDC-3′Cy3-ASO 51 (50, 100 200 µM) dissolved in 0.2 mL of complete high-serum (10% FBS) DMEM medium. Cells incubated in the absence of compounds were considered as negative untreated control. At the end of incubation, 20 µL of MTT (5 mg/mL) in complete high-serum DMEM medium were inserted in each well; then, cells were maintained at 37 °C and 5% CO_2_ for 4 h to allow the conversion by metabolically active cells of the yellow MTT substrate to a purple chromogenic product, formazan. At the end of incubation, the MTT solution was removed, and 0.1 mL were added to each well and maintained in an orbital shaker incubator for 1 h at 37 °C to solubilize the purple MTT formazan crystals. Finally, Sunrise^®^ Microplate Reader (Tecan Trading AG, Männedorf, Switzerland) was set at 570 nm to measure the absorbance of each well. Values, derived from the mean of four independent incubation experiments, were reported as cell vitality percentages with respect to the untreated control incubated in the absence of compounds.

### 2.6. Permeation Studies across Intestinal Cell Monolayers

For the permeation assays, the inserts with the IEC-6 monolayers were washed three times with pre-warmed DPBS buffer in the apical (A, 400 μL) and basolateral (B 2 mL) compartments; DPBS buffer containing 0.9 mM CaCl_2_, 0.5 mM MgCl_2_ and 5 mM glucose at 37 °C was then added to both compartments. ASOs were added to the apical or to the basolateral compartments as ASO 51 or 5′-UDC-ASO 51 100 µM concentrated at apical level, or 50 µM concentrated at basolateral level, to evaluate their permeation. During permeation experiments, inserts were continuously swirled on an orbital shaker (100 rpm; model 711/CT, ASAL, Cernusco, Milan, Italy) at 37 °C. For the A → B influx permeation analysis, 0.4 mL of ASO 51 or 5′-UDC-ASO 51 solutions in DPBS containing salts and 5 mM glucose were added to apical side at time t = 0 and the inserts were placed in the cell culture plate, whose basolateral compartment was prefilled with 2 mL of pre-warmed DPBS with salts and 5 mM glucose. Inserts were moved at the specific timepoints to a subsequent well containing fresh DPBS with salts and 5 mM glucose, allowing harvesting of the previous basolateral DPBS, which was then filtered through regenerate cellulose filters (0.45 µm), and a volume of 10 µL was injected into the HPLC system for the detection and quantification of ASOs. For the B → A efflux permeation studies, the basolateral side of inserts at time t = 0 were filled with 2 mL of ASO solutions in DPBS containing salts and 5 mM glucose, whereas the apical side contained 0.4 mL of fresh DPBS with salts and 5 mM glucose. At fixed time points, the apical samples were removed and replaced with fresh DPBS with salts and 5 mM glucose. The apical samples were filtered through regenerated cellulose filters and injected (10 μL) into the HPLC system for the quantification of ASOs. TEER values were monitored before and after each experiment, as described in Section 2.4. The same experiments were also performed in the absence of cell monolayers, by using cell-free inserts. The concentration values of ASO 51 or 5′-UDC-ASO 51 were expressed as the mean of three independent experiments. Apparent permeability coefficients (P_app_) of ASOs were calculated according to Equation (1) [41,42,43]:

(1) $$P_{\mathrm{app}}=\frac{\frac{\mathrm{dc}}{\mathrm{dt}}{\cdot V}_{r}}{S_{A}\cdot C}$$

with P_app_ representing the apparent permeability coefficient expressed in cm/min; dc/dt, calculated from the slope of the line obtained from the regression of linear data, representing the flux of ASO across the filters of the inserts; V_r_ representing the volume contained in the receiving compartment (basolateral 2 mL); S_A_ representing the diffusion area of the filter (1.12 cm^2^); C representing the ASO concentration in the donor compartment. Papp values were calculated for the filters both in the absence (P_f_) and in the presence (P_t_) of cell monolayers. The P_app_ referred only to the IEC-6 cells’ monolayer (P_E_) and were finally calculated according to Equation (2) [42,44]:

(2) $$\frac{1}{P_{E}}=\frac{1}{P_{t}}-\frac{1}{P_{f}}$$

### 2.7. Exosome Isolation and Characterization

For the isolation of exosomes, ultracentrifugation was used. EXOs were isolated from wild-type 8220 human muscle cell lines (based on the protocol drafted by our collaborator Stephanie Duguez [45]) and fresh cow’s milk. Multiple low-speed centrifugations were performed to progressively remove cells, dead cells and cell debris, followed by filtration through 0.22 and 0.45 μm filters to remove macrovesicles and microvesicles. In the specific case of milk, a treatment with 0.25 mM EDTA for 15 min on ice is carried out to induce milk protein precipitation after performing the primary centrifugation to remove the cells. The purified conditioned medium is then ultracentrifuged at 100,000× *g* for 2.5 h to obtain the exosome pellet, which is subsequently washed three times in PBS to remove residual medium and obtain a purified exosome suspension.

EXOs characterization was carried out by investigating the positivity of exosomal markers (CD63, CD9) and confirming the absence of calnexin using Western Blot (WB). Equal amounts (25 μg) of EVs and cell lysates were boiled with 5x loading buffer and run on NuPAGE^TM^ Bis-Tris Mini Protein Gels (Thermofisher, Milwaukee, WI, USA). Proteins were transferred to Amersham Protran Premium 0.45 μm Nitrocellulose membranes (Sigma, Aldrich, Milan, Italy). Blots were blocked for 1 h at room temperature with 5% Milk-PBS-TWEEN 0.1% for and probed with primary antibodies against CD63 (Sigma), CD9 (Santa Cruz Biotechnology, Dallas, TX, USA), Calnexin (FisherScientific, Milan, Italy), and Vinculine (OZYME, Saint-Cyr-l’Ecole, France) diluted in blocking buffer and incubated overnight at 4 °C. Blots were washed three times with PBS–TWEEN 0.1% and then incubated with IRDye^®^ 800CW Goat anti-Mouse IgG Secondary Antibody (Li-cor, Bad Homburg, Germany) and IRDye^®^ 680RD Goat anti-Rabbit IgG Secondary Antibody (Li-cor, Bad Homburg, Germany). The blots underwent thorough washing three times with PBS–T, followed by detection with Odyssey and quantification using ImageJ (software version 1.49). Furthermore, particle size analyses were conducted with the Zetasizer Ultra Red instrument (Malvern, UK), a combined DLS and ELS system that incorporates Non-Invasive Back Scattering (NIBS) and the exclusive Multi-Angle Dynamic Light Scattering (MADLS) technology to measure particle size and using TEM (EM910 from Zeiss, Jena, Germany) to assess the shape by negative staining. The protocol used involves transferring 10 μL of sample onto a collodion-coated copper grid for 20 min and dried, followed by staining for 10 min with 1% uranyl acetate. The samples were analyzed at 120 kV.

### 2.8. Exosome Loading

A passive strategy, commonly referred to as co-incubation [46], has been employed to load exosomes, enabling the loading of exosomes without affecting their structure. This approach leverages the hydrophobic and lipidic nature of the plasma membrane of exosomes, which facilitates the spontaneous incorporation of cargoes, particularly hydrophobic ones, into exosomes or exosome-secreting cells. The protocol involves the mixture of exosomes and oligos in 100 μL of PBS in a 1:2 ratio for 1.5 h at 37 °C with an oscillation of 500 rpm. For the isolation of loaded and unloaded exosomes, we used the Total Exosome Isolation reagent from cell culture media produced by ThermoFisher, (Milwaukee, WI, USA) following the manufacturer’s protocol. To determine the quantity of oligos loaded in the EXOs, after isolating the loaded or uncharged exosomes as control, we quantified the amount of oligos remaining in the supernatant. We estimated the amount of oligos loaded into the exosomes by subtracting the quantity in the supernatant from the initial quantity added to the mixture.

### 2.9. Lipophilic Membrane Dye Labeling

In order to identify the isolated exosomes and monitor their movement within the target cells, we utilized PKH67 Green Fluorescent Cell Linker Kit for General Cell Membrane Labeling dye (Fischer Scientific, Milan, Italy), according to the manufacturer’s protocol. Briefly, loaded and wild-type exosomes were mixed with DILUENT C to a volume of 1 mL and 6 μL of PKH67 dye. The obtained suspension was mixed constantly for 30 s and then incubated for 5 min at room temperature repaired from light. 2 mL of 10% BSA (A9418) in PBS were added along with FBS-free medium to a total of 8 mL. The suspension is ultracentrifuged for one hour at 4 °C at 100,000× *g* and then resuspended in 5 mL of filtered PBS. The suspension is finally concentrated with Amicon^®^ Ultra-15 100 kDa (Sigma Aldrich, Milan, Italy) columns at 4000× *g* for 5 min to obtain 200 μL of labeled exosome concentrate. Images were collected with the microscope EVOS M5000 (ThermoFisher Scientific, Milan, Italy).

### 2.10. RNA Extraction, Retrotranscritpion, RT–PCR and Exon Skipping Quantification

Total RNA was extracted from myotubes 48 h after the different treatments using the RNeasy Kit (QIAGEN, Hilden, Germany). The RNA was then reverse-transcribed with the High-Capacity cDNA Reverse Transcription Kit (Applied Biosystems, ThermoFisher Scientific, Milan, Italy) as per the manufacturer’s instructions.

RT–PCR was conducted with Platinum Taq (ThermoFisher, Milwaukee, WI, USA) for 28 cycles with primers targeting exon 50 (DMDex50F: CCTGACCTAGCTCCTGGACT) and exon 54 (DMDex54R: GTCTGCCACTGGCGGAGGTC).

One microliter of the RT–PCR product was loaded onto high-sensitivity DNA chips (Agilent, Beijing, China) for quantification of exon skipping. This was done by calculating the ratio of the skipped transcript area to the total area of both skipped and unskipped transcripts, multiplied by 100. All experiments were performed in triplicate.

### 2.11. Non-Contact Co-Culture of DMD Myotubes and Intestinal IEC-6 Cells

Immortalized myoblasts from a Duchenne muscular dystrophy (DMD) patient, were cultured in Skeletal Muscle Growth Medium (Promocell, Heidelberg, Germany). These cells were then induced to differentiate into myotubes by being incubated in Skeletal Muscle Differentiation Medium (Promocell, Heidelberg, Germany) for a minimum of 7 days at 37 °C with 5% CO_2_. Seven days after the start of differentiation of DMD myoblasts into myotubes, IEC-6 cells differentiated for 7 days in tight and polarized monolayers into 12-well inserts (1 µm pore size) were placed in 12-well plates containing myotubes. Skeletal Muscle differentiation medium (2 mL) in the basolateral compartment with myotubes and DMEM containing 1% FBS (0.4 mL) in the apical compartment with IEC-6 cells were conditioned for 24 h to define the relationship between muscle and intestinal cells under co-culture conditions. Thereafter, to evaluate if 5′-UDC-3′Cy3-ASO 51 can cross the intestinal barrier of IEC-6 cells and affect DMD myotubes, it was applied overnight at 37 °C and 5% CO_2_ into the apical compartment at the final concentration of 100 µM as free compound (gymnosis) or loaded in exosomes obtained from milk (MilkEXOs) in DMEM plus 0.1% bovine serum albumin (BSA). Indeed, exosomes normally present in FBS can interfere with exogenous milk-exosomes, thus previously reported options for medium depletion of FBS-derived exosomes include replacing FBS in the basal culture medium, supplemented with all nutrients and antibiotics, with BSA to optimize cell survival [47]. Mono-cultured DMD myotubes were incubated with MilkEXOs (10 µM 5′-UDC-3′Cy3-ASO 51) as positive control. TEER of IEC-6 cell monolayers was measured before and after the incubation period with 5′-UDC-3′Cy3-ASO 51 and compared to untreated IEC-6 cell monolayers, alone or co-cultured with DMD myotubes. At the end of the incubation with 5′-UDC-3′Cy3-ASO 51, the apical incubation medium was withdrawn and stored at 4 °C until the final quantification of 5′-UDC- 3′Cy3-ASO 51 by NanoDrop 1000 Spectrophotometer (ThermoFisher Scientific, Milan, Italy). Myotubes in the bottom of the wells were separated from the IEC-6 cells in the inserts, maintained for 48 h at 37 °C and 5% CO_2_, and analysed for their intake of the 5′-UDC-3′Cy3-ASO 51. Mono-cultured myotubes incubated with MilkEXOs (10 µM 5′-UDC-3′Cy3-ASO 51) were also maintained for 48 h at 37 °C and 5% CO_2_. The basolateral incubation medium was withdrawn and stored at 4 °C until the final quantification of 5′-UDC-3′Cy3-ASO 51 by NanoDrop 1000 Spectrophotometer (ThermoFisher Scientific, Milan, Italy). Untreated DMD myotubes co-cultured with IEC-6 cell monolayers were used as negative control for both 5′-UDC-3′Cy3-ASO 51 and exon skipping quantification. The experiment was performed in triplicate. The scheme of the co-culture procedure and images of the equipment used are shown in Figure 2.

### 2.12. Statistical Analyses

Statistical analyses were performed using Graph Pad Prism software, version 7 (GraphPad Software, San Diego, CA, USA). Statistical comparisons between apparent permeability coefficients (P_E_) values or their ratios were performed by one-way analysis of variance (ANOVA) followed by Tukey’s multiple comparisons post-tests, or by t-Student test. Statistical comparisons between cell viability in the MTT assay were performed by ANOVA followed by Dunnett’s multiple comparisons test. Significant difference was set at *p* < 0.05.

Statistical analyses for exosome loading comparison and exon skipping experiments were performed using Graph Pad Prism software, version 8.0.1 (GraphPad Software, San Diego, CA, USA). Statistical comparisons between the different loading studies values or their ratios were performed by paired *t*-test and one-way ANOVA followed by two-way ANOVA multiple comparisons. Statistical comparisons between different exon skipping results were performed by paired *t*-test and one-way ANOVA. Significant difference was set at *p* < 0.05.

### 2.13. Microscopy and Image Elaboration

Fluorescent images were collected using EVOS M5000 (ThermoFisher Scientific, Milan, Italy), an inverted cell imaging system for four-color fluorescence and transmitted-light applications. All the images were captured with the same exposure time and were exported to tiff files that were analyzed using the ImageJ software 1.49 (National Institutes of Health, Bethesda, MD, USA).

## 3. Results and Discussion

### 3.1. IEC-6 Cell Viability

**Figure 3:**
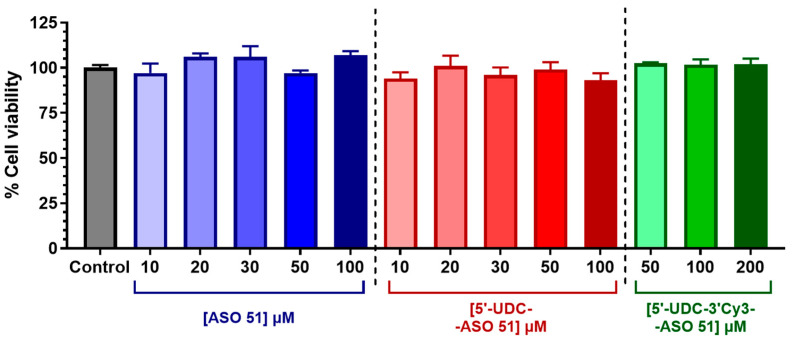
Cell viability of IEC-6 cells incubated overnight with different concentrations of ASO 51 (shades of blue), 5′-UDC-ASO 51 (shades of red) or 5′-UDC-3′Cy3-ASO 51 (shades of green). Results are reported as cell viability percentage (%) normalized to untreated control (in the absence of compounds). Data are expressed as mean ± S.E.M. of four independent experiments and were statistically analyzed by one-way ANOVA followed by Dunnett’s multiple comparisons test, showing no statistically significant differences in cell viability in the presence of compounds compared to untreated control.

Since the viability of intestinal cells is a fundamental requisite to guarantee the integrity of the cell monolayer during permeation experiments, the potential cytotoxic effects of increasing concentrations of ASO 51, 5′-UDC-ASO 51 and 5′-UDC-3′Cy3-ASO 51 were firstly evaluated by a standard (1-(4,5-dimethylthiazol-2-yl)-3,5-diphenylformazan) MTT assay. MTT is a colorimetric assay based on the reduction of a yellow substrate (tetrazolium salt, MTT) to a purple product (formazan) by mitochondrial dehydrogenase of metabolically active cells, and it is commonly used to detect potential toxicity of compounds in IEC-6 cells [48,49]. The amounts of formazan produced are proportional to the number of viable cells. Figure 3 reports the results about the cell viability of IEC-6 incubated overnight with increasing concentrations of ASO 51, 5′-UDC-ASO 51 or 5′-UDC-3′Cy3-ASO 51. In particular, ASO 51 and 5′-UDC-ASO 51 did not affect the cell viability of IEC-6 cells up to 100 µM concentration. Moreover, no toxic effects were evidenced after incubation of 5′-UDC-3′Cy3-ASO 51 up to 200 µM concentration. About the potential effects of exosomes on cell viability, it is known that MilkEXOs do not affect the cell viability of Caco-2 cells after 24 h of incubation [50]. This behaviour matches with our results describing the TEER values of IEC-6 monolayers incubated with 5′-UDC-3′Cy3-ASO 51 loaded in MilkEXOs. The details of these results are reported in Section 3.7.

### 3.2. Permeation Studies across Intestinal Monolayers

To evaluate the potential ability of ASOs to permeate across intestinal barriers, we performed in vitro permeability studies across monolayers obtained by IEC-6 cells. These cells are an established, non-transformed cell line derived from primary normal epithelial cells of the rat small intestine [29]. Other intestinal barrier models of tumor origin, such as Caco-2 cells, are characterized by loss of contact inhibition and polarization, resulting in changes in growth and mono- and multilayer formation [51]. On the other hand, the IEC-6 cell line preserves almost all the physiological properties of the small intestine, resulting in suitability for permeability studies. IEC-6 cells form a polarized monolayer in semi-permeable inserts, expressing the features of an epithelial intestinal barrier, separating two different compartments: the upper compartment, which represents the apical side (A) of the cells, corresponds on the side of the monolayer facing the intestinal lumen; the lower compartment, representing the basolateral side (B) of the cells, corresponds to the side of the monolayer facing the bloodstream [30]. With this system, the permeation of ASOs across the intestinal barrier can be easily simulated in vitro. The accumulated concentrations (μM) in the receiving compartment over time (Figure S3) allowed us to obtain linear fits whose slopes were used to calculate, according to Equation (1), the permeability coefficient (P_t_ and P_f_), in turn used in Equation (2) to obtain the final apparent permeation coefficient (P_E_) of ASOs, referring to the IEC-6 cell monolayer only (Section 2.6). As reported in Figure S3, the cumulative amounts of ASO 51 or 5′-UDC-ASO 51 in the receiving compartments during time evidenced linear profiles (r ≥ 0.998, *p* ≤ 0.001) at all tested conditions (i.e., in the absence or presence of cell monolayers, from apical to basolateral compartments and vice versa). These cumulative patterns also indicate a lower permeation of the ASOs in the presence of the confluent IEC-6 cells on the filters of cellQART inserts. In particular, the overlapped chromatograms reported in Figure S4 constitute a representative indication of this behaviour, which confirms the ability of the cell monolayers to behave as a physiological barrier, according to the TEER values measured at confluence for the IEC-6 monolayers of about 50 Ω·cm^2^, as expected for this type of cell line [52].

**Figure 4:**
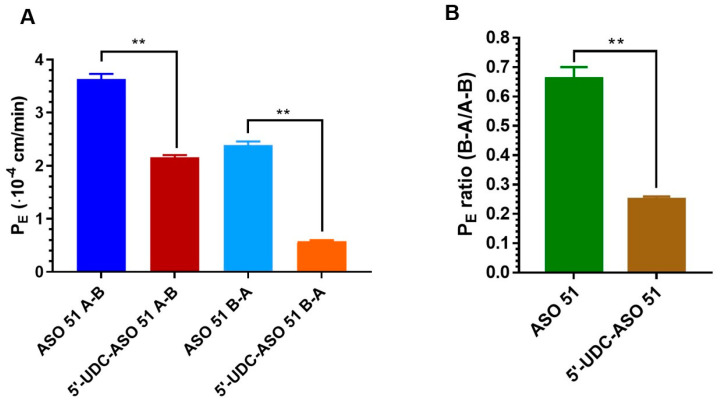
(**A**) Permeation coefficients (P_E_) of ASO 51 and 5′-UDC-ASO 51 across the intestinal monolayer of IEC-6 cells from apical to basolateral compartments (**A**–**B**) and vice versa (**B**–**A**). All data are reported as the mean ± S.E.M of three independent experiments. (**B**) P_E_ ratio between efflux (**B**–**A**) and influx (**A**–**B**) permeation of ASO 51 and 5′-UDC-ASO 51 across the intestinal monolayer of IEC-6 cells. The values are reported as the mean ± S.E.M. of three independent experiments. ** *p* < 0.0001.

Permeability studies were therefore performed to evaluate the bidirectional permeation of ASO 51 or 5′-UDC-ASO 51 across the polarized IEC-6 cell monolayers in cellQART inserts for a 12-well plate. The values of the apparent permeability coefficients (P_E_) of each ASO are represented in Figure 4A, where the P_E_ values refer to the permeation from the apical to the basolateral compartments (A→B, basolateral as receiving compartment) and vice versa (B→A, apical as receiving compartment) for ASO 51 and 5′-UDC-ASO 51.

The P_E_ values represented in Figure 4A evidence the ability of the ASOs to permeate across the intestinal monolayers in both directions. In particular, for the A→B direction, the P_E_ values for ASO 51 and 5′-UDC-ASO 51 were (3.63 ± 0.10)∙10^−4^ cm/min and (2.16 ± 0.04)·10^−4^ cm/min, respectively, and for the B→A direction (2.39 ± 0.07) 10^−4^ cm/min and (0.54 ± 0.01)∙10^−4^ cm/min, respectively. These data indicate that UDCA conjugation to ASO 51 appears to slightly, but significantly (*p* < 0.0001), decrease the permeation rates in both directions.

We had previously reported a similar decrease for the antiviral drug zidovudine (AZT) after its conjugation with UDCA. Indeed, the prodrug UDCA–AZT, resulting from this conjugation, was characterized by P_E_ values across intestinal cell monolayers significantly lower than those of AZT [53]. This reduction was attributed to the increase in AZT molecular weight upon conjugation, enhanced from 267.24 Da to 641.80 Da. It is, indeed, known that molecules above 500 Da face difficulties in crossing physiological barriers [54,55]. However, these concerns do not apply to ASO 51 and 5′-UDC-ASO 51 because of their molecular weight, which is already too high (6991.80 and 7633.66 Da, respectively) to allow their diffusion across the bilayers of the cellular membranes; moreover, the molecular weight enhancement of ASO 51 due to the UDCA conjugation appears negligible in comparison to its overall molecular weight. Yet, in contrast with ASO 51, 5′-UDC-ASO 51 evidences an aptitude to rearrange itself in aqueous media, leading to supramolecular aggregates, which have been identified as self-assembled nanoparticles with a size of around 150 nm, significantly higher than the 2 nm size attributed to the single molecules [25]. This phenomenon, evidenced for lipid conjugates of macromolecules [56], can be related to the P_E_ decrease across intestinal monolayers of ASO 51 induced by its conjugation with UDCA. It is, in any case, interesting to compare the P_E_ ratio values between the B→A and A→B directions, obtained for the investigated ASOs. In particular, as represented in Figure 4B, these ratio values appear significantly different (*p* < 0.001), at 0.66 ± 0.04 for ASO 51 and 0.25 ± 0.01 for 5′-UDC-ASO 51. In particular, the P_E_ ratio value of less than 0.5 for 5′-UDC-ASO 51 denotes the aptitude of this compound to experience a resultant influx from the apical to basolateral compartments [57], which in our system simulate in vitro the intestinal lumen and the bloodstream, respectively.

According to this point of view, we have previously evidenced that a P_E_ ratio lower than 0.5 for the in vitro permeation of geraniol across intestinal cell monolayers is related to a very high oral bioavailability of this compound [58]. This information suggests that the 5′-UDCA conjugation to ASO 51 should improve its potential oral bioavailability, as the P_E_ ratio of 5′-UDC-ASO 51 is significantly lower than that of its parent compound.

In reinforcement of this point of view, it can be observed that the conjugation of AZT with UDCA obtained the compound UDCA–AZT, able to elude active efflux transporters of AZT [53]. As a consequence, despite the lower permeation rate of UDCA–AZT across physiological barriers [53], its uptake in murine macrophages appeared more than 20 times greater than that of AZT [59]. Moreover, a self-aggregated nanoparticle of UDCA–AZT, obtained by nanoprecipitation, evidenced an uptake in murine macrophages up to 70 times higher in comparison to the dissolved compound [60]. The size of the UDCA–AZT nanoparticles was about 200 nm [60], very similar to that of the self-assembled 5′-UDC-ASO 51 nanoparticles, whose main population evidenced a size of about 150 nm [25]. Taking into account this information, it is possible to hypothesize that the aptitude of 5′-UDC-ASO 51 to self-aggregate as nanoparticles can induce in intestinal cells endocytosis or macro-pinocytosis mechanisms for its transport from the intestinal lumen to the bloodstream, which may be advantageous for oral bioavailability. It is, in fact, known that cell uptake of antisense oligonucleotides is predominantly mediated by endocytosis [61] and that the transport mechanisms of nanoparticles across intestinal tissue can offer new oral delivery perspectives [62].

### 3.3. Exosome Isolation and Characterization

**Figure 5:**
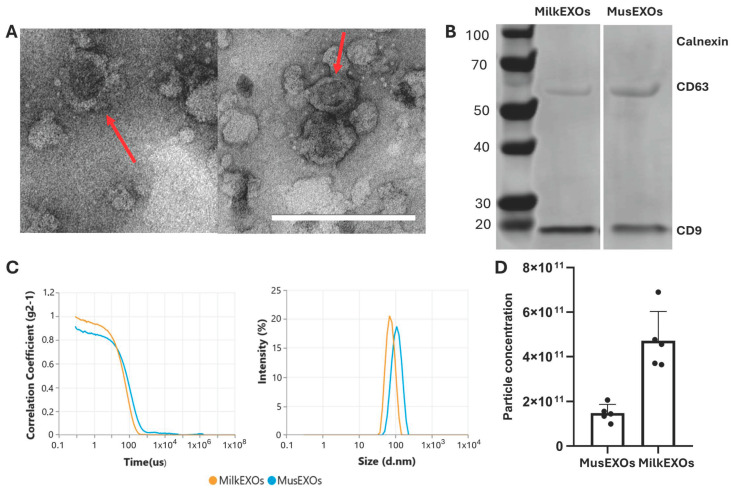
Exosome characterization. (**A**) Morphological characterization of isolated exosomes was carried out using TEM, (**left**) MusEXOs, (**right**) MilkEXOs. Scale bar: 200 nm. Red arrows indicate exosomes. (**B**) Western blot analysis of exosome-specific markers (CD9 and CD63) and the endoplasmic reticulum marker (calnexin) was performed from exosomal protein extracts. (**C**) Size distribution of isolated exosomes measured using Zetasizer Ultra red instrument (Malvern Panalytical). The analysis produced a correlogram (**left**) and showed a distribution in the size of isolated exosomes with defined peaks and absence of polydispersity (**right**). (**D**) Particle concentration was evaluated by particle count, using the Zetasizer Ultra Red Instrument (Malvern Analytical). Each dot represents a different extraction of exosomes from the same initial amount of sample.

To evaluate the potential of exosomes to deliver antisense oligonucleotides, we isolated exosomes (EXOs) from immortalized human muscle cell lines (MusEXOs) and fresh cow’s milk (MilkEXOs). To confirm effective isolation, we analyzed the size and shape of the nanovesicles using the Zetasizer Ultra Red instrument and transmission electron microscopy (TEM). Isolated nanovesicles were round, as seen in Figure 5A, with dimensions of about 100 nm for exosomes isolated from the immortalized human muscle cell line and 89 nm for those isolated from fresh cow’s milk (Figure 5B). Western Blot analysis confirmed the presence of specific exosomal markers CD63 and CD9, which are commonly associated with exosomes, and the absence of calnexin, an endoplasmic reticulum protein marker, which serves as a negative control (Figure 5A). To further assess the quality of our preparations, we analyzed sample dispersion and particle quantity. The results showed one-dimensional populations in all cases, with a polydispersity index below 0.25, indicating high homogeneity and purity of samples, with a high number of particles per sample (Figure 5C).

### 3.4. Exosomes Are Efficiently Loaded with Both 5′-UDC-3′Cy3-ASO 51 and 3′Cy3-ASO 51

The loading of exosomes with our ASOs was evaluated with the co-incubation technique, experiments conducted by varying the volume of co-incubation and the quantity of ASOs and exosomes in order to maximize the loading efficiency.

**Figure 6:**
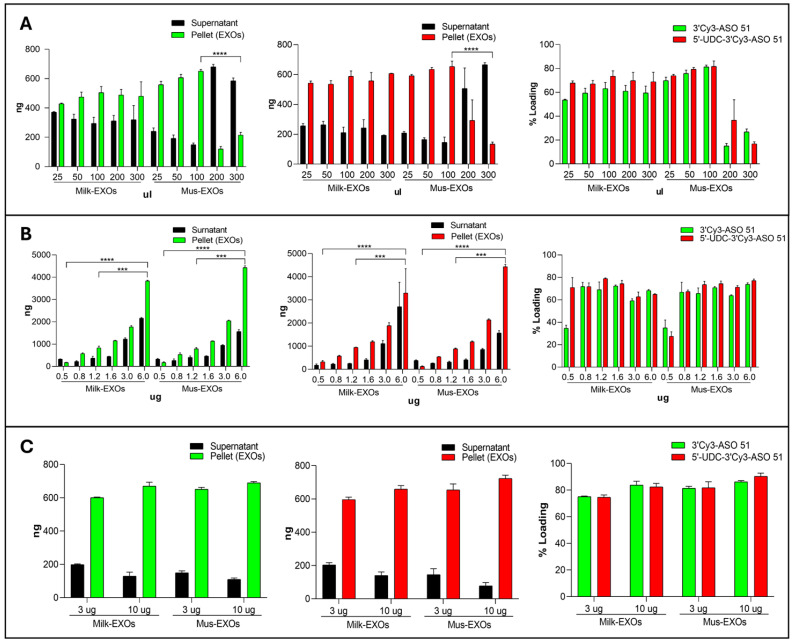
Co-incubation loading studies to identify the best ratio of ASOs and EXOs to achieve the highest loading efficiency. (**A**) Evaluation of the effect of different co-incubation volumes on loading efficiency. Different volumes (25, 50, 100 200 and 300 μL) of PBS were tested by mixing 3 μg of Milk-EXOs and Mus-EXOs with 800 ng of 5′-UDC-3′Cy3-ASO 51 (red) or 3′-Cy3-ASO 51 (green); (**B**) Evaluation of the effect of different amounts of ASOs on loading efficiency: 3 μg of Milk-EXOs and Mus-EXOs were mixed with 500, 800, 1200, 1600, 3000 and 6000 ng of 5′-UDC-3′Cy3-ASO 51 (red) or 3′Cy3-ASO 51 (green) for a ratio of 6:1, 3.75:1 2.5:1, 1.87:1, 1:1 and 1:2; (**C**) Evaluation of the effect of different quantity of EXOs on loading efficiency: 3 and 10 μg of Milk-EXOs and Mus-EXOs with 800 ng of 5′-UDC-3′Cy3-ASO 51 (red) or 3′Cy3-ASO 51 (green) in 100 μL of filtered 1X PBS. SUR-NATANT: ASOs quantified in the supernatant (BLACK); PELLET: ASOs quantified in the pellet, then loaded into the exosomes (GREEN: 3′Cy3-ASO 51; RED 5′-UDC-3′Cy3-ASO 51). Values are expressed as the mean ± standard deviation (SD) of three experiments. *** *p* < 0.0002 paired *t*-test, ordinary one-way ANOVA and two-way ANOVA multiple comparisons; **** *p* < 0.0001 paired *t*-test, ordinary one-way ANOVA and two-way ANOVA multiple comparisons.

We first investigated several co-incubation volumes by mixing 3 μg of exosomes isolated from fresh cow’s milk (Milk-EXOs) or immortalized muscle cells (Mus-EXOs) with 800 ng of 5′-UDC-3′Cy3-ASO 51 or 3′Cy3-ASO 51 in 25, 50, 100 200, and 300 μL of filtered PBS (Figure 6A) We achieved a comparable loading efficiency across various co-incubation volumes for exosomes isolated from fresh cow’s milk. However, for exosomes isolated from immortalized muscle cells, a drastic reduction in loading efficiency was observed when the volume exceeded 100 μL.

We then examined the impact of different amounts of ASOs added to the co-incubation suspension on loading efficiency. We combined 3 μg of Milk-EXOs and Mus-EXOs with 500, 800, 1200, 1600, 3000, and 6000 ng of 5′-UDC-3′Cy3-ASO 51 or 3′Cy3-ASO 51 (to obtain ratios of 6:1, 3.75:1 2.5:1, 1.87:1, and 1:1). An increase in loading efficiency correlated with an increase in the amount of ASOs added to co-incubation with both types of exosome (Figure 6B).

Lastly, we evaluated the contribution of exosome quantity to loading efficiency. We combined 3 and 10 μg of Milk-EXOs and Mus-EXOs with 800 ng of ASOs in 100 μL of filtered PBS and observed a similar loading efficiency in both cases (Figure 6C). The optimal loading efficiency was obtained by using a ratio of 2 μg of ASO per 1 μg of exosomal protein, resulting in the loading of 3.8 μg of 3′Cy3-ASO 51 and 4.4 μg of 5′-UDC-3′Cy3-ASO 51 for 3 μg of exosomes. Notably, the conjugated ASO was loaded in slightly higher quantities than the ASO51 (Figure 6B), likely due to its lipophilic nature and enhanced ability to form nanoparticles compared to its unconjugated counterpart [25].

### 3.5. EXO–ASO Complexes Efficiently Fuse to Myotubes, Distributing ASOs in the Cytoplasms, but Not in the Nucleus

It is known in the literature that exosomes can fuse with target cells by acting as media or transporters [33]. Exosomes offer several benefits over other delivery systems, such as viral vectors and nanoparticles. They are naturally occurring, biocompatible, and less immunogenic, reducing the risk of adverse immune responses. Exosomes have an inherent ability to facilitate cellular uptake and deliver therapeutic cargo across biological barriers, enhancing the delivery efficiency of drugs and genetic material. However, their limitations include the complexity of isolation and purification processes, potential for unintended cargo delivery, and scalability challenges for large-scale production. Despite these challenges, exosomes hold significant promise for targeted and efficient drug delivery.

**Figure 7:**
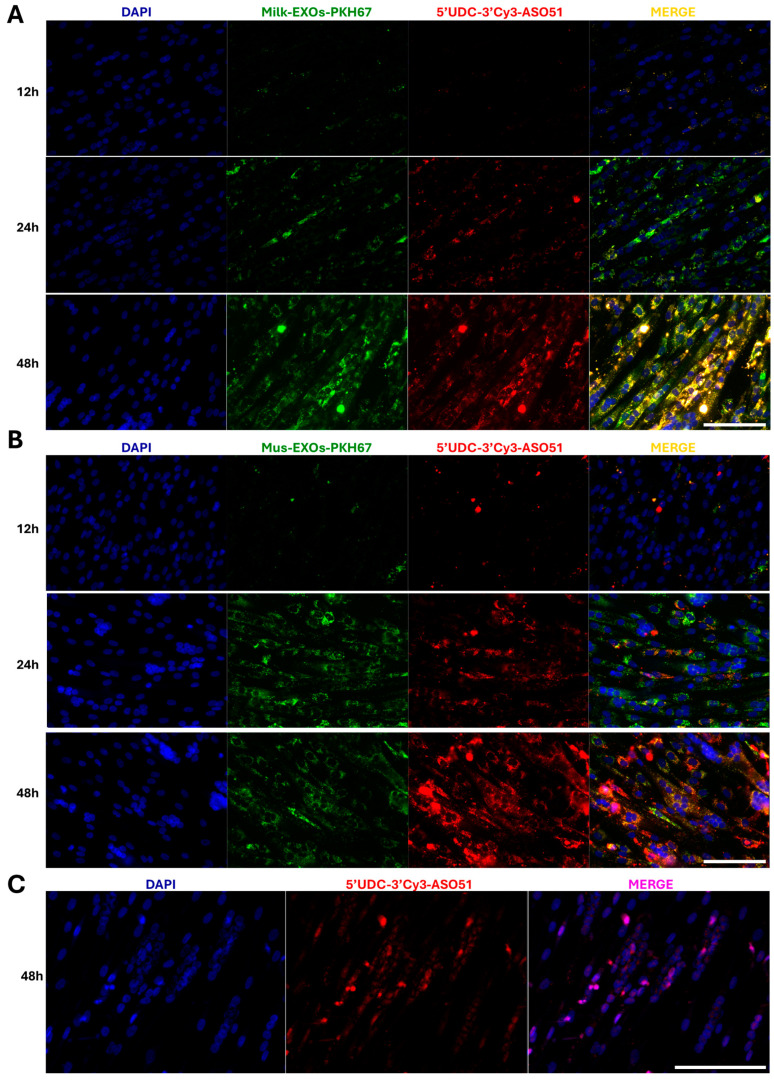
EXOs-ASOs complexes fusion and distribution studies. Exosomes isolated from MilkEXOs (**A**) and MusEXOs (**B**) labelled with the fluorescent dye PKH67 (green) and loaded with 5′-UDC-3′Cy3-ASO 51 (red) were administered to KM1328 myotubes. The ability of EXOs–ASOs complexes to fuse with target cells and deliver ASOs was evaluated at different time points (12, 24 and 48 h). DAPI: Nucleus (blue). (**C**) 5′-UDC-3′Cy3-ASO 51 (red) was transfected with JetPEI into KM1328 myotubes. DAPI: Nucleus (blue). Scale bar: 75 μm.

Related to this, Milk-EXOs and Mus-EXOs were labeled with the fluorescent green dye PKH67, loaded with 5′-UDC-3′Cy3-ASO 51, and then administered to myotubes differentiated from immortalized myoblasts with a deletion of exon 52, with the aim of confirming the ability to fuse with target cells and assessing the capacity of delivery ASOs. We monitored the fusion of complexes with cells at various time points (12, 24 and 48 h) to track their distribution. The results revealed that fluorescence was predominantly distributed within cells and exhibited a significant increase with time in both cases (Figure 7A,B), indicating the potential for exosomes to merge with target cells.

The analysis showed a strong colocalization between PKH67 and Cy3, especially after 48 h, confirming the ability of the exosomes to transport the ASOs inside the muscle cells.

However, we observed limited nuclear fusion, as ASOs predominantly accumulated in the cytoplasm of cells. In contrast, the transfection-related result using the JetPei transfecting agent led to strong nuclear accumulation (Figure 7C). This result is related to the renowned ability of polyethylenimine (PEI) to facilitate endosomal escape, a crucial process for the efficient release of nucleic acids within cells, making JetPEI as an extremely effective carrier for nucleic acid delivery [63]. To increase the number of ASOs carried by EXOs that reach the nucleus, the use of compounds that facilitate endosomal escape or nuclear localization signals could be exploited [64]. Such elements could enhance the release of oligonucleotides into the cytoplasm and uptake into the nucleus, thereby increasing the efficacy of the treatment. By integrating these endosomal escape compounds into EXOs–ASOs gene delivery formulations, the amount of genetic material reaching the nucleus of target cells could be optimized, enhancing their efficacy in therapeutic applications.

### 3.6. MusEXO-ASO Complexes Induce Higher Levels of Exon Skipping in Myotubes Compared to MilkEXO-ASO Complexes and Gymnotic Delivery

To assess the potency of Mus- and Milk- EXOs-ASOs complexes to induce exon 51 skipping, we added these complexes directly to KM1328 myotubes. For comparison across different conditions, we delivered 2 µg of 3′Cy3-ASO 51 or 5′-UDC-3′Cy3-ASO 51 either alone (gymnotic delivery) or loaded in 20 µg of Mus- and Milk- EXOs. JetPEI transfecting agent was used as positive control for delivery.

The results revealed a skipping rate of 91.5% with 5′-UDC-3′Cy3-ASO 51 and 85% with 3′Cy3-ASO 51 in JetPEI transfected myotubes. Compared to our previous studies, the exon skipping efficiency of the 3′Cy3-ASO 51 is much higher than the ASO 51 (less than 10%, [25]) suggesting that Cy3 might act similarly to UDCA in improving the efficacy of ASOs by increasing its lipophilicity. This is also true for the 5′,3′-bis-UDC-ASO 51 as, without the Cy3, this reached roughly 70% skipping [25]. This may also explain the almost equal loading in the exosome of the two ASOs used in this study.

**Figure 8:**
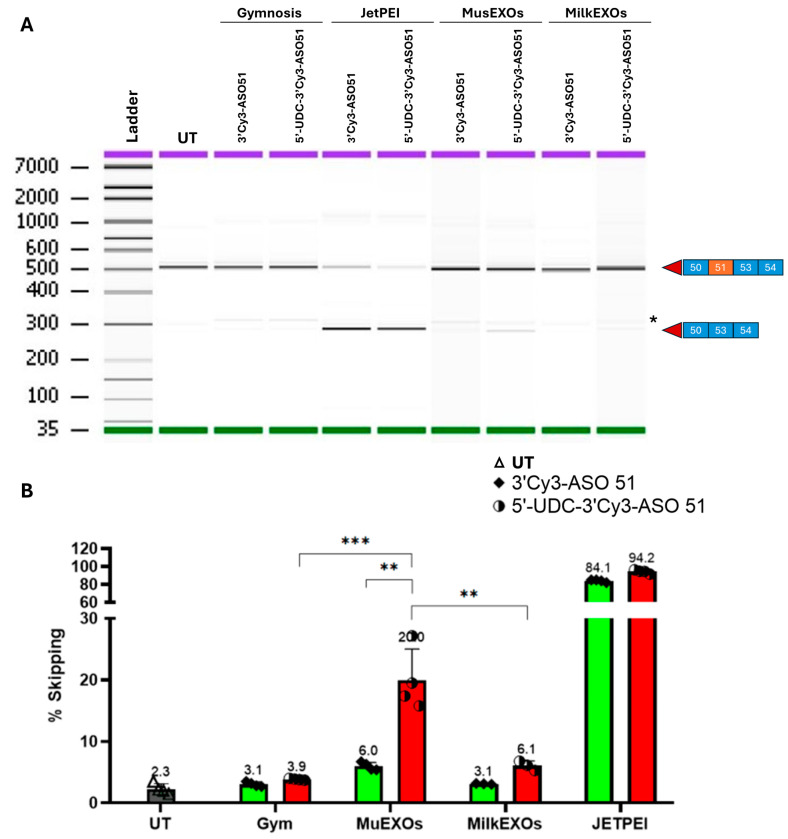
Exon skipping analysis in KM1328 myotubes treated with Mus- or Milk-EXOs-ASOs complexes compared to gymnosis delivery and JetPEI transfection. (**A**) Representative gel capillary electrophoresis: on the right side of the image, the squares represent the exon composition of the corresponding bands of 514 bp for the out-of-frame transcript that includes exon 51 and 281 bp for the skipped transcript. Purple: Upper marker; green: Lower marker. * Unspecific band highlighted in each sample, including UT. (**B**) Exon skipping results: the quantification of exon skipping was performed by determining the percentage ratio, calculated as the area of the skipped transcript divided by the total area of both skipped and unskipped transcripts, multiplied by 100. UT: Untreated; Gym: Gymnosis. Values are expressed as the mean ± standard deviation (SD) of four experiments. ** *p* < 0.001 paired *t*-test and ordinary one-way ANOVA; *** *p* < 0.0002 paired *t*-test and ordinary one-way ANOVA.

The skipping efficiency of MusEXOs-5′-UDC-3′Cy3-ASO 51 complexes was 20%, compared to 6% achieved with MusEXOs-3′Cy3-ASO 51 complexes. Only 6.1% of skipping was revealed with MilkEXOs-5′UDC-3′Cy3-ASO 51 complexes compared to 3.1% achieved with MilkEXOs-3′Cy3-ASO 51 complexes. Gymnotic delivery induced 3.9% and 3.1% with 5′-UDC-3′Cy3-ASO 51 and 3′Cy3-ASO 51, respectively (Figure 8B).

These findings show that administration of MusEXOs-5′-UDC-3′Cy3-ASO 51 complexes led to a three-fold increase in skipping efficiency compared to its unconjugated counterpart and to MilkEXOs-5′-UDC-3′Cy3-ASO 51 complexes, and a five-fold increase compared to gymnotic delivery. Considering the almost similar skipping efficiency of the two ASOs, any significant difference in these results should be addressed regarding the exosomes’ properties.

These results confirmed the ability of loaded exosomes to fuse with myotubes and deliver the ASOs. However, MusEXOs were more effective in inducing exon skipping compared to MilkEXOs and gymnotic delivery. This is not surprising, as exosomes derived from particular tissues exhibit a higher tendency to fuse with the same tissue type [65].

### 3.7. Non-Contact Co-Culture of IEC-6 and DMD Muscle Cells: 5′-UDC-3′Cy3-ASO 51 after Crossing the IEC-6 Monolayers Can Induce Exon Skipping in Muscle Cells

The results of the experiments reported in Section 3.2 highlighted the ability of 5′-UDC-ASO 51 to cross the intestinal barrier in vitro and, in particular, the presence of a potential influx transport, in contrast with ASO 51. Moreover, the data reported in Section 3.5 indicate the aptitude of exosomes to deliver ASOs to DMD muscle cells. Based on these results, non-contact co-culture between IEC-6 cells and DMD myotubes was performed in order to evaluate 5′-UDC-ASO 51’s potential ability, either as free compound (gymnotic delivery) or loaded in MilkEXOs, to cross the intestinal IEC-6 monolayer, enter DMD myotubes and induce exon skipping. The non-contacting cell co-culture system allowed the two cell lines to be cultured separately at different times and in two different compartments (insert membrane and well), allowing, once put together, a bidirectional diffusion of soluble factors through the pore of the insert membrane [66].

Wolf and collaborators demonstrated that the use of MilkEXOs can be considered a good strategy to improve the delivery of microRNAs across the intestinal barrier because of an active endocytotic transport from apical to basolateral compartments in the IEC-6 cell monolayer, confirming that the upper small intestine was the main site of milk-exosomes’ absorption [67].

In our case, the experiment was performed by incubating 5′-UDC-ASO 51 conjugated to a fluorescent probe, i.e., 5′-UDC-3′Cy3-ASO 51, since the presence of the Cy3 dye was necessary to localize the ASO inside intestinal and muscle cells.

The results of permeation studies reported in Section 3.2 showed that a 3-h incubation in A→B direction with 100 µM 5′-UDC-ASO 51 induced a cumulative concentration of about 2 µM in the basolateral compartment, which is far from the 25 µM concentration previously applied to DMD myotubes in gymnotic delivery, to obtain a significant exon skipping [25]. Therefore, a concentration of 100 µM (about 1 mg/mL) of 5′-UDC-3′Cy3-ASO 51 as free compound or loaded in MilkEXOs (about 1 mg/mL of ASO) was applied overnight to the apical side of IEC-6 cells previously co-cultured for 24 h with differentiated DMD myotubes in the bottom of the plate. As stated above (Section 2.11), to deplete the medium of FBS-derived exosomes and avoid cross interactions with the milk-exosomes, 0.1% BSA was added instead of 1% foetal bovine serum (FBS) [47] to preserve the integrity of the plasma membrane in the cell monolayer.

**Figure 9:**
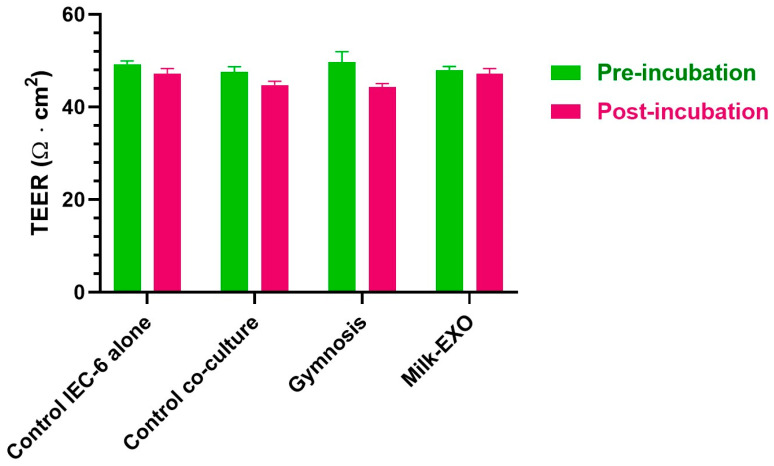
TEER measurement of IEC-6 cell monolayer co-cultured for 24 h with myotubes measured before (“pre-incubation”) and after (“post-incubation”) the incubation with 5′-UDC-3′Cy3-ASO 51 in gymnotic delivery (“Gymnosis”) or as MilkEXO (“MilkEXO”). IEC-6 cell monolayer alone or co-cultured 24 h with myotubes were used as negative controls. Data are expressed as the mean ± S.E.M. of three independent experiments. No significant differences were observed.

To confirm that the presence of DMD myotubes does not alter the integrity of the cell monolayers, TEER measurements were performed before and after the incubation with 5′-UDC-3′Cy3-ASO 51 in gymnotic delivery or loaded in MilkEXO, as reported in Figure 9. In particular, no significant differences in TEER values of IEC-6 cell monolayers were observed in the presence or the absence of DMD myotubes at the bottom of the plate for 24 h, and neither before nor after overnight incubation with 5′-UDC-3′Cy3-ASO 51 in gymnotic delivery or loaded in MilkEXOs, showing values of about 50 Ω·cm^2^, as expected for this type of cell line [52]. These results are in good agreement with those obtained by previous studies, evidencing the unaffected viability of Caco-2 cells incubated for 24 h with bovine loaded MilkEXOs [50].

TEER-based assay of viability is indeed considered a suitable indicator of the health of both barrier tissue models and co-culture of multiple cell types such as IEC-6 cell monolayers and myotubes, as evidenced in other co-culture models [68].

After the overnight incubation, the medium containing 5′-UDC-3′Cy3-ASO 51 or MilkEXOs loaded with the same compound was withdrawn from the apical compartments of IEC-6 cells and replaced with 1% FBS DMEM medium.

**Figure 10:**
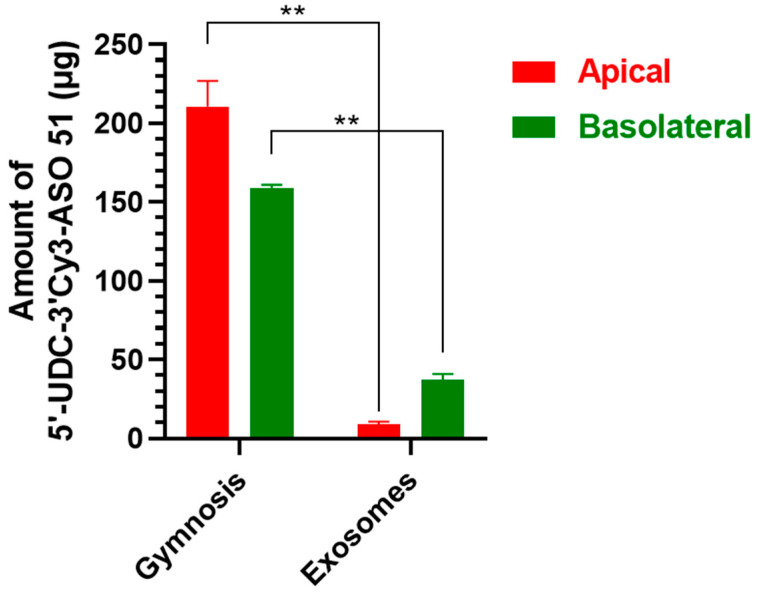
Amounts of 5′-UDC-3′Cy3-ASO 51 expressed in µg contained in 0.4 mL of medium for the apical compartment and 2 mL of medium for the basolateral compartment, quantified 48 h after the end of the co-culture experiment. Data are expressed as the mean ± S.E.M. of three independent experiments. ** *p* < 0.01 in the comparison of the same compartment in different incubation conditions.

Figure 10 reports the amounts of 5′-UDC-3′Cy3-ASO 51 quantified by NanoDrop Spectrophotometry at the end of the experiment in the co-culture conditions, applying 100 µM 5′-UDC-3′Cy3-ASO 51 (400 µg) on the apical side of the system in gymnotic delivery or as MilkEXOs.

In gymnotic conditions, 210.44 ± 16.33 µg of compound were detected in the apical compartment (0.4 mL), and 158.87 ± 1.98 µg in the basolateral compartment (2 mL), indicating a loss of about 30 µg of ASO, presumably internalized and/or metabolized into IEC-6 and/or DMD cells. In MilkEXOs loaded conditions, only 8.81 ± 1.96 µg were detected in the apical compartment (0.4 mL), which is significantly lower compared to the amount quantified in gymnotic delivery (*p* < 0.01). Similarly, only 37.19 ± 3.81 µg were detected in the basolateral compartment (2 mL), in this case also significantly lower than that quantified in the same compartment in gymnotic delivery (*p* < 0.01). The total loss of ASO was about 354 µg, one order of magnitude higher than that observed in gymnotic delivery, indicating a higher internalization and/or metabolism of compound in intestinal or muscle cells.

When MilkEXOs loaded with 5′-UDC-3′Cy3-ASO 51 were added directly to DMD cells at a concentration of 10 µM (200 µg), only 35.89 ± 5.81 µg were detected in the incubation medium (2 mL), suggesting that more than 80% of the compound was internalized and/or metabolized into the muscle cells. Therefore, these results indicate that MilkEXOs are significantly transported by IEC-6 cells and internalized mainly by DMD myotubes.

**Figure 11:**
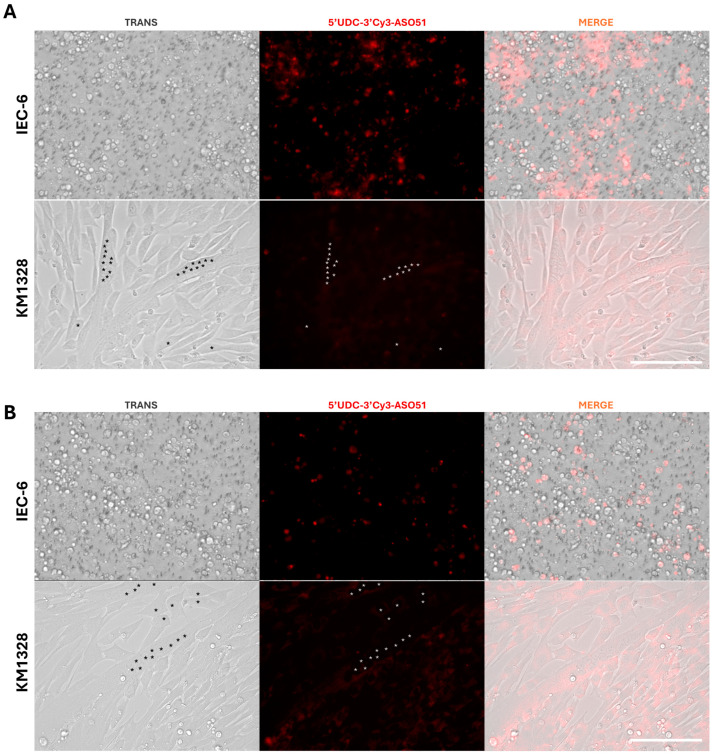
Fluorescence distribution of 5′-UDC-3′Cy3-ASO 51 into the IEC-6 and KM1328 myotubes after 48 h of gymnotic delivery (**A**) or incapsulated into MilkEXOs (**B**). With *, we highlight the nuclei of the myotubes. Scale bar: 75 μm.

The active transport of ASOs from the apical to the basolateral compartment through intestinal cells was confirmed via microscopy by visualizing the fluorescence in the basolateral compartment. High concentrations of free ASOs were observed in the basolateral compartment, showing significantly higher levels in gymnosis treatment compared to MilkEXOs treatment (Figure 10). In addition, visualization of fluorescent ASOs revealed accumulation within the endoplasmic reticulum and membranes of intestinal cells, demonstrating uptake through both gymnotic and exosomal administration (Figure 11) with higher quantities for gymnosis. Traces of ASOs were also detected within muscle cells: a higher concentration of fluorescence was observed in myotubes treated with MilkEXOs-5′-UDC-3′Cy3-ASO 51 complexes compared to their gymnotic counterparts (Figure 11).

To evaluate the efficacy of exon skipping in the KM1328 myotubes at the bottom of the basolateral compartment, cells were collected after 48 h of treatment and RNA extracted and retrotranscribed to perform exon skipping analysis.

**Figure 12:**
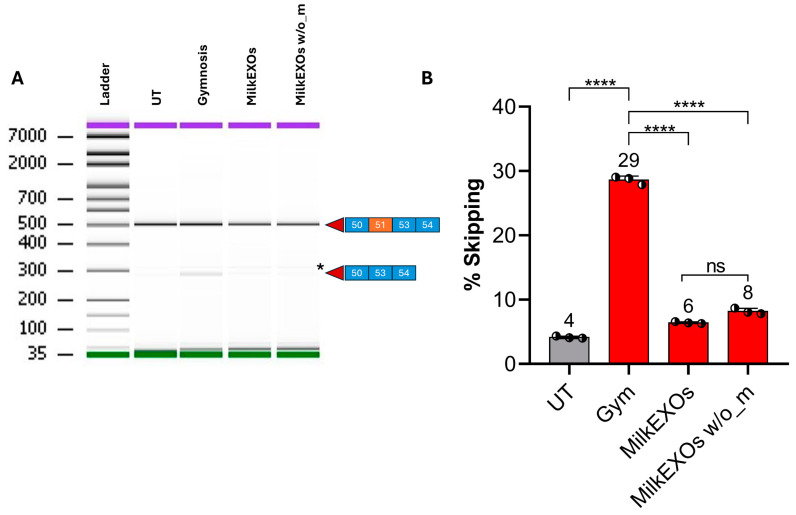
Exon skipping analysis in in vitro permeation studies. (**A**) Representative gel capillary electrophoresis: on the right side of the image, the squares represent the exon composition of the corresponding bands as described in Figure 8. Purple: Upper marker; green: Lower marker. * Unspecific band highlighted in each sample, including UT. (**B**) Exon skipping quantification. UT: Untreated; Gym: Gymnosis. MilkEXOs w/o_m: exon skipping results from the direct administration of MilkEXOs-5′-UDC-3′Cy3-ASO 51 to the myotubes. Values are expressed as the mean ± standard deviation (SD) of three experiments. **** *p* < 0.0001 (paired *t*-test and one-way ANOVA).

The exon skipping analysis from the myotubes confirmed that the levels of ASOs in the basolateral compartment were enough, after the permeation across the IEC-6 cell monolayer, to induce exon skipping, resulting in a skipping efficiency of 6% with MilkEXOs-ASO complex and a surprising 29% obtained with the gymnotic administration of 5′-UDC-3′Cy3-ASO 51 (Figure 12B). Untreated non-contact co-cultures of IEC-6 cells and myotubes were used as negative control (UT, Figure 12B), similarly to myotubes treated directly with MilkEXOs (MilkEXOs w/o_m, Figure 12B). These controls indicate comparable results, ranging from 4% to 6% skipping efficiency values, which appear significantly lower (*p* < 0.0001) than the skipping efficiency on DMD myotubes obtained by the gymnotic administration of 5′-UDC-3′Cy3-ASO 51 in the apical compartment of IEC-6 cell monolayers (29%).

Taken together, these results suggest that the MilkEXOs-ASO 51 complexes can cross the intestinal monolayer and fuse with myotubes at the bottom of the basolateral compartment, but the ASO does not reach the nucleus in quantities high enough to induce significant levels of skipping (Figure 12B).

These findings suggest a divergence from previous microscopy data, where myotubes treated with MilkEXOs carrying 5′-UDC-3′Cy3-ASO 51 exhibited notably higher fluorescence compared to their gymnotic counterparts. This increased fluorescence intensity suggests enhanced uptake and retention of ASOs delivered via MilkEXOs through the intestinal monolayer. In contrast, exon-skipping assays revealed that myotubes treated with free 5′-UDC-3′Cy3-ASO 51, after the permeation across the intestinal monolayer, exhibited a five-fold higher exon-skipping percentage compared to those treated with milk-derived exosome complexes.

This is, indeed, interesting if we consider that, after the permeation across the intestinal monolayer and just before exon skipping measurements (48 h after the end of the co-culture experiment), we detected a 5′-UDC-3′Cy3-ASO 51 concentration of about 9 μM (150 μg in 2 mL of medium) in the basolateral compartment. This concentration value is 2.7 times lower than that used in our previous work [25], in which 5′-UDC-ASO 51 was delivered by gymnosis at 25 μM concentration and resulted in roughly more than 6% of exon skipping.

The result showed in Figure 12B for the free 5′-UDC-3′Cy3-ASO 51 may be explained by the potential production of exosomes by intestinal cells. These exosomes could efficiently deliver the ASO to the nucleus of DMD myotubes.

In fact, rat intestinal epithelial cells, like IEC-6, are recognized for their ability to release exosome-like vesicles crucial for intercellular communication and the transfer of bioactive molecules, including microRNAs [69,70,71].

In our study, a similar phenomenon could be at play: the substantial presence of free ASOs observed within intestinal cells under microscopy suggests that these ASOs might be incorporated into exosomes during their formation. These exosomes are then released into the basolateral compartment, where they can fuse with muscle cells. This process is supported by previous findings, which demonstrated that exosomes derived from IEC-6 cells can indeed fuse with target cells [71].

The observed differences in exon-skipping efficacy might be due to variations in the bioactive molecules within exosomes derived from intestinal cells compared to those from MilkEXOs. Intestinal cells might load their exosomes with specific components that enhance the delivery of ASOs to muscle cell nuclei, potentially improving the endosomal escape process.

Moreover, within exosomes formed by intestinal cells, substances may accumulate that induce differential solute content between nascent endosomes and the cytoplasm. This accumulation can lead to endosomal destabilization and subsequent ASO release [72]. Further investigation is warranted to fully elucidate these mechanisms and optimize exosome-based therapies.

## 4. Conclusions

In conclusion, we demonstrated that 5′-UDC-ASO 51 permeates the intestinal monolayer with a potential influx mechanism more efficient in comparison to ASO 51. Moreover, we evidenced that the permeation across the intestinal barrier does not restrain the ability of 5′-UDC-3′Cy3-ASO 51 to induce remarkable levels of skipping in muscle cells but, on the contrary, the intestinal barrier crossing strongly enhances this ability.

Moreover, we demonstrated that exosomes can be easily loaded with antisense oligonucleotides for exon skipping and that loaded MusEXOs can induce higher skipping than gymnotic delivery when directly administered to target cells. However, neither gymnotic nor exosome delivery are comparable to JetPEI transfection, which facilitates endosomal escape and preferential accumulation of ASOs within the nucleus of myotubes and extremely high exon skipping in vitro.

This suggest that further investigations are needed to improve the delivery of ASOs with exosomes. In fact, MilkEXOs failed to efficiently deliver the 5′-UDC-3′Cy3-ASO 51 to myotubes’ nuclei, either directly or through the intestinal monolayer, and accumulated in the cytoplasm.

## Data Availability

The original contributions presented in the study are included in the article/Supplementary Materials, further inquiries can be directed to the corresponding author.

## Supplementary Materials

The following supporting information can be downloaded at: https://www.mdpi.com/article/10.3390/pharmaceutics16081023/s1, Figure S1: Representative chromatogram obtained by the HPLC analysis of 5 μM ASO 51 dissolved in DPBS; Figure S2: Representative chromatogram obtained by the HPLC analysis of 5 μM 5′-UDC-ASO 51 dissolved in DPBS; Figure S3: Cumulative amounts of ASO 51 or 5′-UDC-ASO 51 in the receiving compartments after permeation across cellQART inserts in the absence (filter) or in the presence of polarized IEC-6 cell monolayers (cells); Figure S4: Representative overlapped chromatograms obtained by the HPLC analysis of 5′-UDC-ASO 51 after 1-h permeation across cellQART inserts from apical to basolateral compartments in the absence (filter) or in the presence of polarized IEC-6 cell monolayers (cells); Figure S5: Unprocessed western blot analysis of exosomes-specific markers (CD9 and CD63) and the endoplasmic reticulum marker (calnexin) reported in Figure 5B of the main text.
