# Supplementary material for: In Vitro Studies to Evaluate the Intestinal Permeation of an Ursodeoxycholic Acid-Conjugated Oligonucleotide for Duchenne Muscular Dystrophy Treatment
Source: Pharmaceutics. 2024 Aug 1;16(8):1023. doi: 10.3390/pharmaceutics16081023 (PMC11360444; doi:10.3390/pharmaceutics16081023)

In Vitro Studies to Evaluate the Intestinal Permeation of an Ursodeoxycholic Acid-conjugated Oligonucleotide for Duchenne Muscular Dystrophy Treatment

Marika Faiella[1†], Giada Botti[2,3†], Alessandro Dalpiaz[2], Lorenzo Gnudi[4], Aurélie Goyenvalle[5], Barbara Pavan[6,3*], Daniela Perrone[4*], Matteo Bovolenta[1§] and Elena Marchesi[2§]

[1] Department of Translational Medicine, University of Ferrara, 44121 Ferrara, Italy; fllmrk@unife.it (M.F.); bvlmtt@unife.it (M.B.)

[2] Department of Chemical, Pharmaceutical and Agricultural Sciences, University of Ferrara, 44121 Ferrara, Italy; bttgdi@unife.it (G.B.); dla@unife.it (A.D.); mrclne@unife.it (E.M.)

[3] Center for Translational Neurophysiology of Speech and Communication (CTNSC@UniFe), Italian Institute of Technology (IIT), 44121 Ferrara, Italy

[4] Department of Environmental and Prevention Sciences, University of Ferrara, 44121 Ferrara, Italy; lorenzo.gnudi@unife.it (L.G.)

[5] Université Paris-Saclay, UVSQ, Inserm, END-ICAP, 78000 Versailles, France; aurelie.goyenvalle@uvsq.fr (A.G.)

[6] Department of Neuroscience and Rehabilitation—Section of Physiology, University of Ferrara, 44121 Ferrara, Italy

* Correspondence: prd@unife.it (D.P.); pvnbbr@unife.it (B.P.)
† Co-first authors
§ Co-last authors

Figure S1. Representative chromatogram obtained by the HPLC analysis of 5 μM ASO 51 dissolved in DPBS.

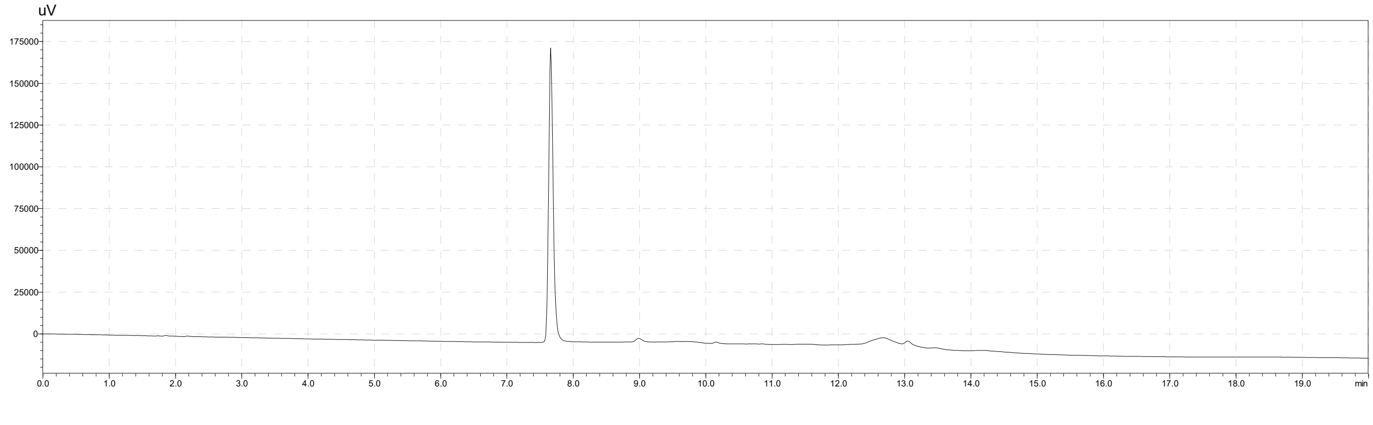

Figure S2. Representative chromatogram obtained by the HPLC analysis of 5 μM 5'-UDC-ASO 51 dissolved in DPBS.

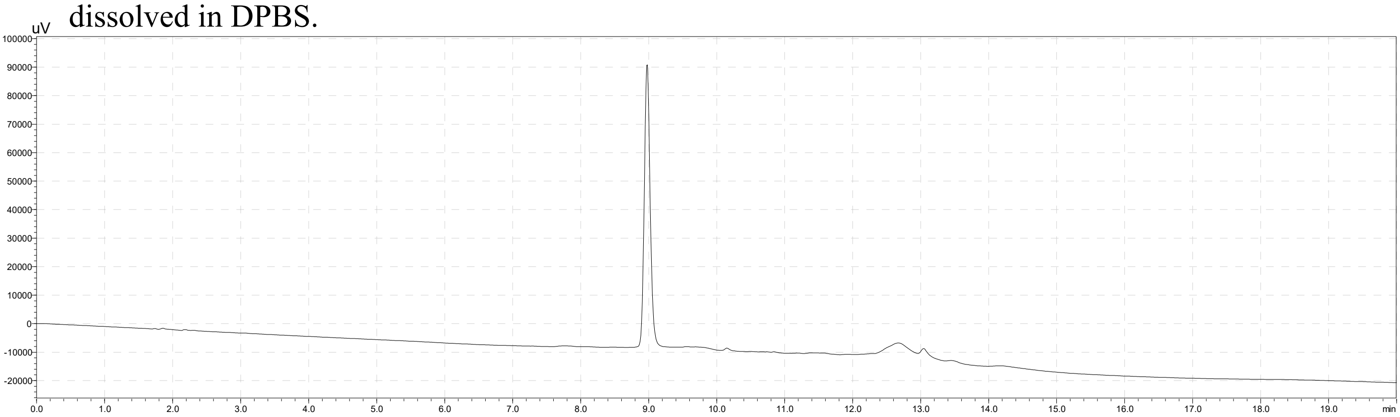

Figure S3. Cumulative amounts of ASO 51or 5'-UDC-ASO 51 in the receiving compartments after permeation across cellQART inserts in the absence (filter) or in the presence of polarized IEC-6 cell monolayers (cells). The permeations were performed from apical to basolateral compartments of the cellQART system (A→B) and viceversa (B→A). All data are reported as the mean ± S.E.M of three independent experiments.

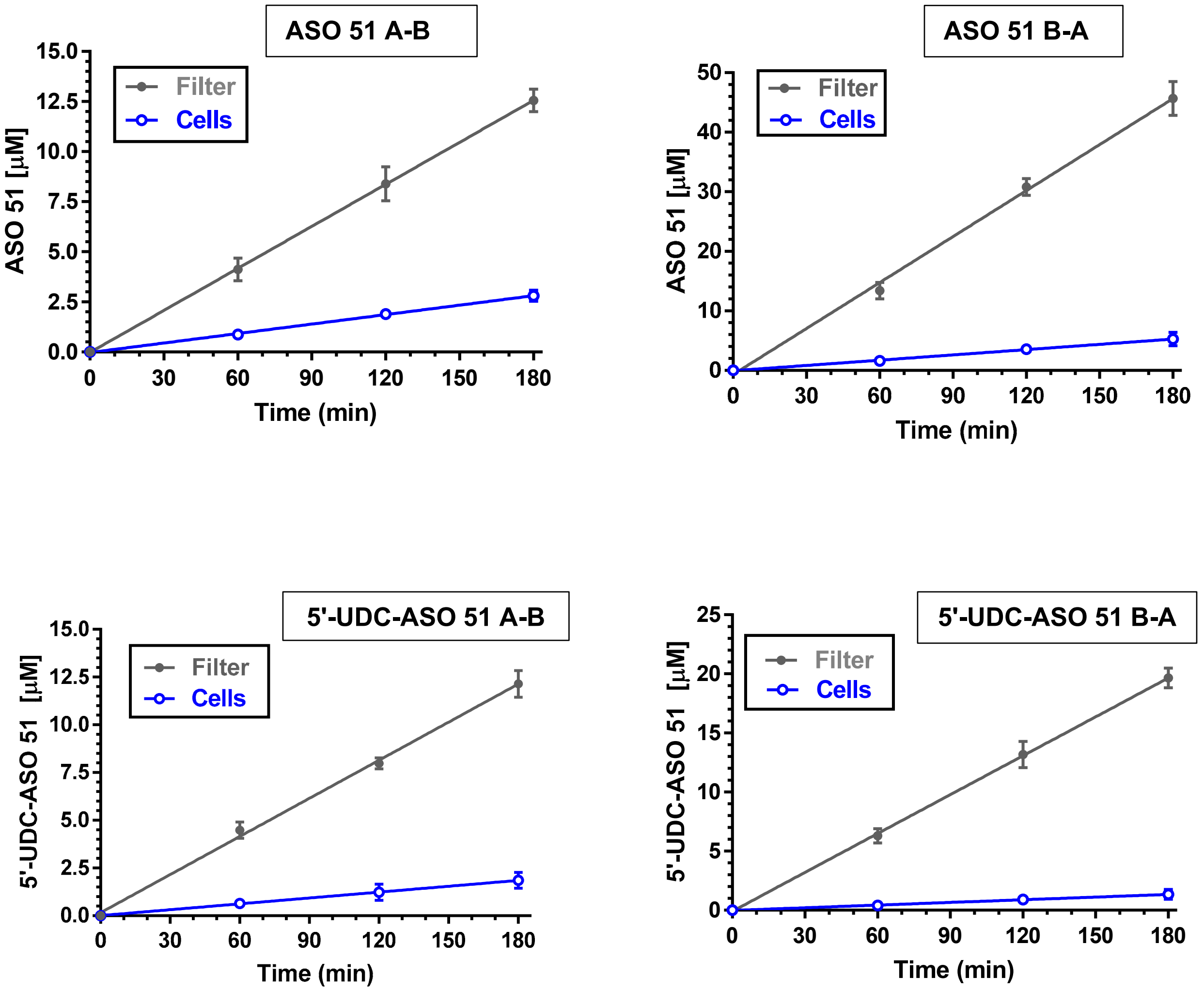

Figure S4. Representative overlapped chromatograms obtained by the HPLC analysis of 5'-UDC-ASO 51 after 1-hour permeation across cellQART inserts from apical to basolateral compartments in the absence (filter) or in the presence of polarized IEC-6 cell monolayers (cells).

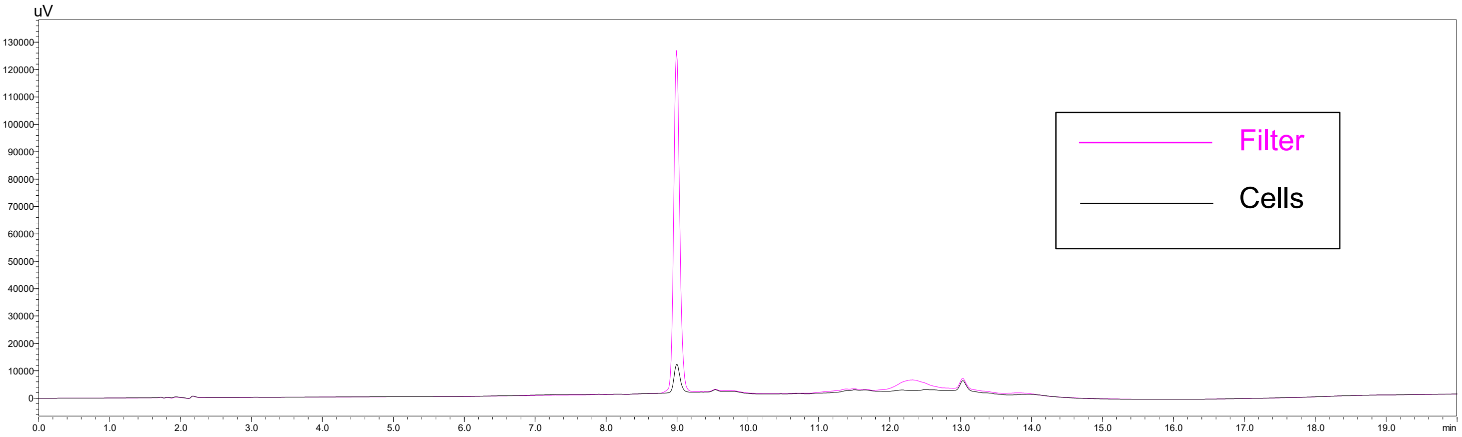

Figure S5. Unprocessed Western blot analysis of exosomes-specific markers (CD9 and CD63) and the endoplasmic reticulum marker (calnexin)

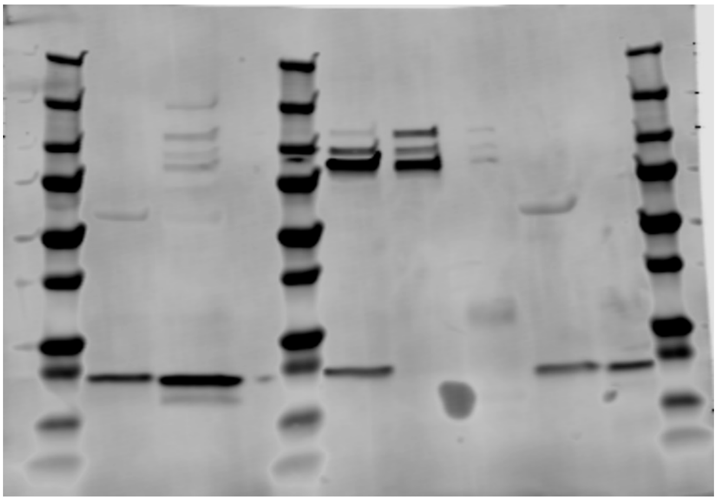

Supplement: Supplementary file 1 [file pharmaceutics-16-01023-s001.zip › pharmaceutics-3115369-supplementary.pdf]
